# Case Report: Intravitreal dexamethasone implant as adjuvant treatment for taxane-related cystoid macular edema

**DOI:** 10.3389/fopht.2022.972623

**Published:** 2022-08-18

**Authors:** Xujia Liu, Xiaohua Ding, Guoqiao Lin

**Affiliations:** ^1^ Joint Shantou International Eye Center of Shantou University and the Chinese University of Hong Kong, Shantou, China; ^2^ Postgraduate Department, Shantou University Medical College, Shantou, China

**Keywords:** taxanes, paclitaxel, cystoid macula edema, optical coherence tomography, fluorescein angiography, intravitreal dexamethasone implant

## Abstract

Taxane-related cystoid macular edema (CME) is a rare complication of the taxoid medication, a chemotherapeutic drug. We report a 47-year-old Han Chinese man referred to our Eye Center for decreased vision with visual distortion in both eyes for two weeks. Two weeks prior, he received the last cycle of his six-monthly chemotherapy, including paclitaxel for hypopharyngeal malignancy. The best-corrected visual acuity (BCVA) was 0.4 OD and 0.1 OS. Macular optical coherence tomography showed significant bilateral CME, and fluorescein angiography (FA) revealed the fluorescein pooling at the late phase without leakage. Intravitreal 700 μg dexamethasone (DEX) implant was applied to the left eye and 13 days after to the right eye. Two months later, the macular morphology recovered to normal. One year after the first visit, the BCVA was 1.0 OD and 0.8 OS with standard macula on OCT. In conclusion, the intravitreal DEX implant might be an effective adjuvant treatment for taxane-related CME.

## Introduction

Taxanes, a class of anticancer drugs, were divided into three types: paclitaxel (PTX), marketed as Taxol®, Anzatax®, or Paxene®; the synthetic derivative of PTX, including docetaxel (DTX) (Taxotere®) and cabazitaxel (CTX) (Jevtana®); and nanoparticle albumin-bound (nab) paclitaxel (Abraxane®). Taxanes stabilize the intracellular microtubules, a highly dynamic cytoskeletal structure to inhibit mitosis and other vital cellular functions, such as cell movement, intracellular transport, and differentiation (1). These antimitotic characteristics make it an indication of cancers.

Taxane-related cystoid macular edema (CME) is a rare complication of the taxoid medication and always affects both eyes. The reported incidence rate range from 0.198% to 0.5% (2, 3). The onset of CME varies from two months to a half years after taxane administration (4). Taxane-related CME is a self-limiting disease recovered after cessation of taxane treatment. The recovery time varies largely from 2 weeks to 24 weeks, which primarily depends on the severity of the CME. Severe CME with highly elevated central macular thickness typically takes longer to recover, which may cause irreversible damage to the macular function. Various treatments have been tried to relieve the CME and accelerate recovery, including topical NSAID, topical dorzolamide, topical steroids, and intravitreal anti-VEGF (3). In this case report, we demonstrate a case recovered from taxane-related CME by cessation of taxoid with intravitreal dexamethasone implant (DEX).

## Materials and methods

The uncorrected visual acuity (UCVA) and best-corrected visual acuity (BCVA) were measured by a standard visual acuity chart and recorded by decimal acuity. The ILM-RPE thickness was used to represent the central macular thickness (CMT), measured automatically by spectral-domain optical coherence tomography (SD-OCT) (Cirrus HD-OCT Model 4000; Zeiss, Jena, Germany) and divided into three areas according to the ETDRS grid: the foveal is the central 1 mm diameter of the ETDRS grid; the perifoveal is the inner macular ring of the ETDRS grid (diameter range from 1 mm to 3 mm), and the perifoveal is the outer macular ring of the ETDRS grid (diameter 3 mm to 6 mm). The parafoveal and perifoveal were subdivided into four areas: superior, inferior, nasal, and temporal. The dots represent the mean of ILM-RPE thickness in 4 regions, and the shadows represent the 95% confidence interval of the mean. The line plot and statistics were calculated and drawn by Python package Seaborn 0.11.2 and Matplotlib 3.5.2. Details of the anonymous OCT reports can be obtained from: https://github.com/XujiaLiu/dex_for_taxane_induced_cme.

## Case presentation

**Figure 1:**
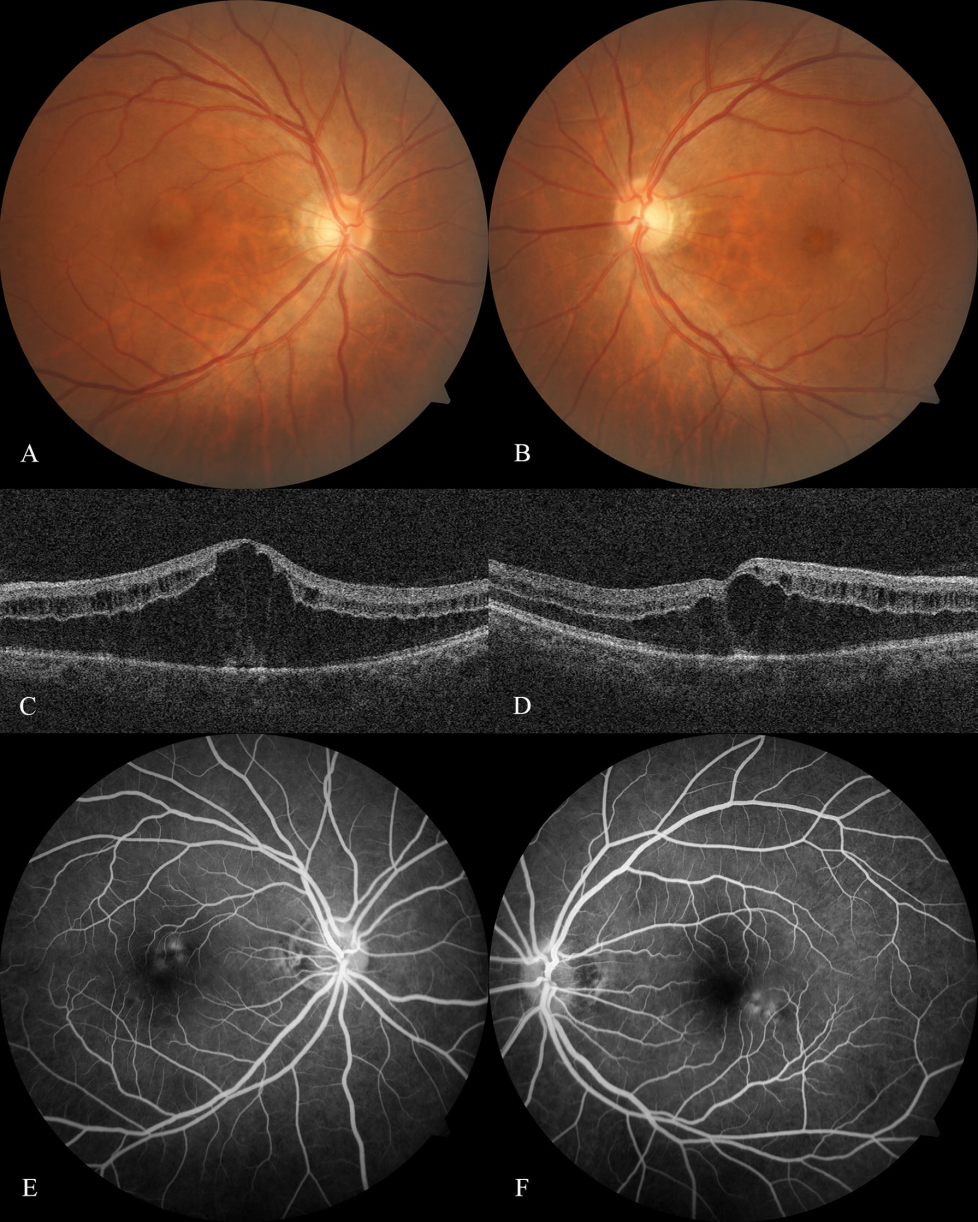
Optical coherence tomography and fluorescein angiography at baseline Fundus photography of the right eye **(A)** and left eye **(B)** at baseline showed the honeycomb-like CME pattern. Optical coherence tomography scan of the right eye **(C)** and left eye **(D)** at baseline revealed a substantial elevation of Henle's fiber layer and cystoid-like edema scattered within the inner retinal layer. Fluorescein angiography at baseline revealed the fluorescein-stained fluid superior to the right eye fovea **(E)** and inferior to the left eye **(F)** starting at the retinal venous phase, resulting in the late pooling of a characteristic flower petal pattern and no leakage had been shown.

A 47-year-old Han Chinese man presented with decreased vision with visual distortion in both eyes for two weeks. Two weeks prior, he had the last cycle of his six-monthly chemotherapy for hypopharyngeal malignancy, including 200 mg pembrolizumab, 100 mg cetuximab, 30 mg cis-platinum, and 100 mg albumin-bound paclitaxel. Albumin-bound paclitaxel is the only drug reported responsible for CME. No similar symptoms have been shown in his previous treatments. On examination, the UCVA was 0.12 OD and 0.08 OS, and the BCVA was 0.4 OD and 0.1 OS. External slit-lamp examination showed mild opacity of the bilateral lens. Dilated fundus examination and fundus photography revealed a bilateral honeycomb-like CME pattern (**Figures 1A, B**). The macular optical coherence tomography (OCT) confirmed the fundus examination findings of CME (**Figures 1C, D**). Fluorescein angiography (FA) revealed the fluorescein pooling at the late phase without leakage (**Figures 1E, F**). Intravitreal 700 μg dexamethasone (DEX) implant (Ozurdex, Allergan, Irvine, CA) was administered to the left eye, and an intense follow-up was scheduled to monitor macular responses for DEX.

**Figure 2:**
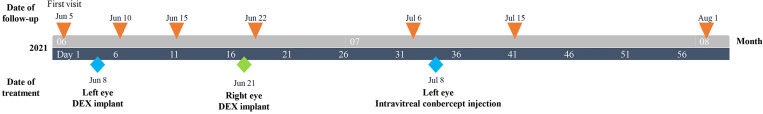
Clinical timeline for a 47-year-old Chinese male patient with taxane-related cystoid macular edema.

**Figure 3:**
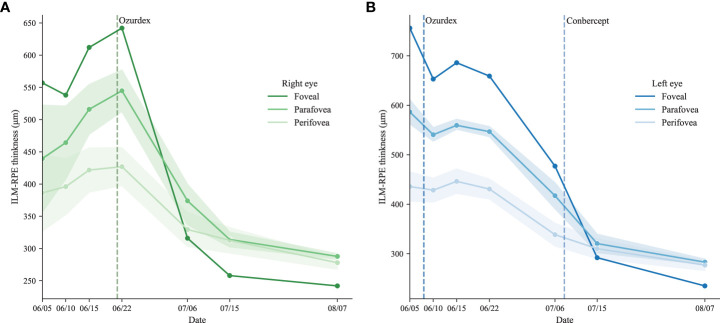
The trend of ILM-RPE thickness The ILM-RPE thickness of the ETDRS grid for the right eye **(A)** and the left eye **(B)**.

**Figure 4:**
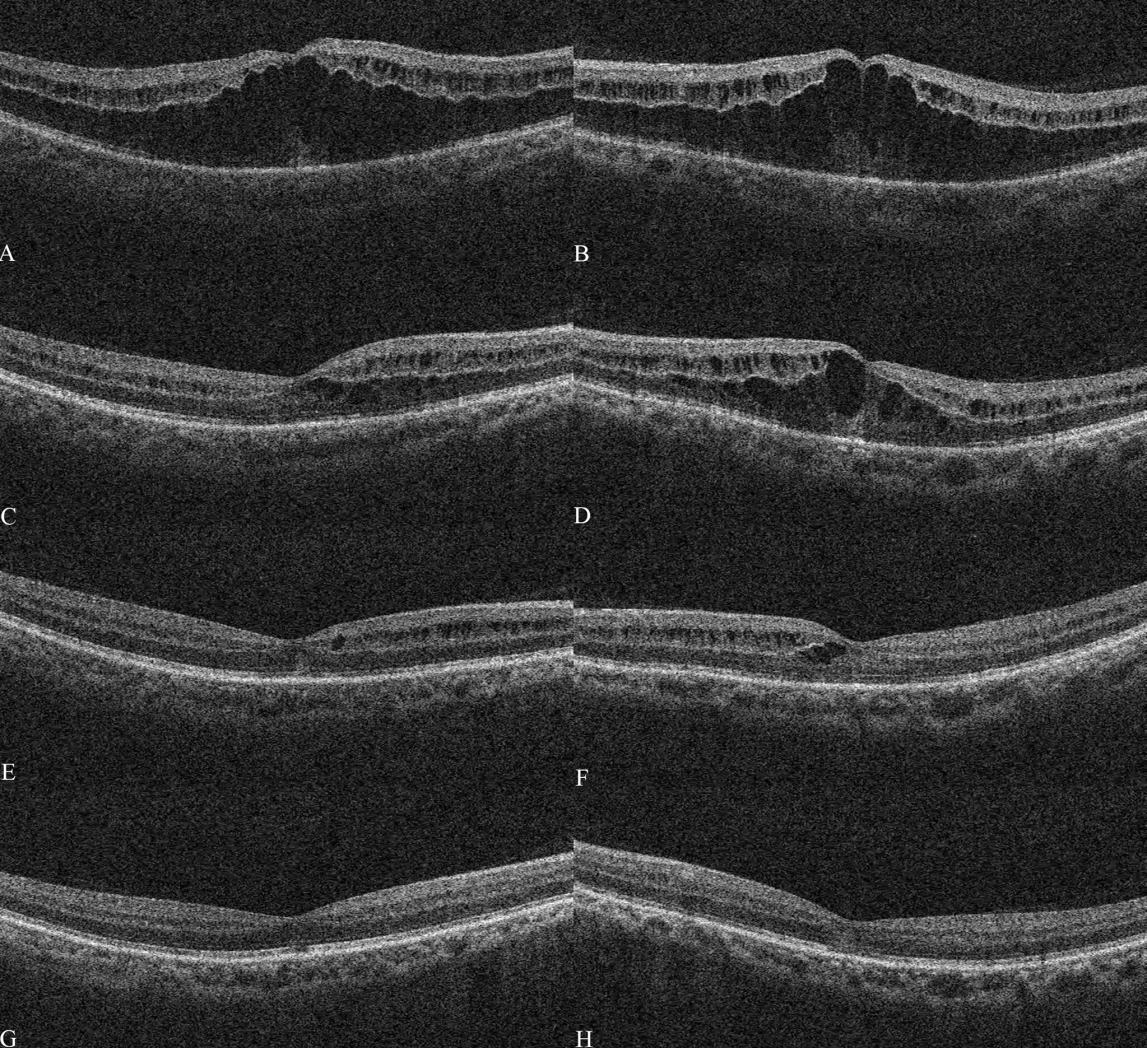
Optical coherence tomography after treatment The macular optical coherence tomography (OCT) scans showed macular morphology of the right eye and left eye on Jun 18^th^ **(A, B)**, July 6^th^ **(C, D)**, July 15^th^ **(E, F)**, and Aug 7^th^ **(G, H)**.

The patient was guided to follow up on day 3, week 1, and 13 days postoperatively (**Figure 2**). The UCVA improved to 0.15 for the left eye and slightly improved from 0.12 to 0.15 for the right one. OCT showed that the central subfield thickness (CST) decreased from 756 μm to 644 μm for the left eye, and the CST of the untreated eye increased from 557 μm to 628 μm (**Figures 3A, B**). The right eye was then administered with an intravitreal DEX implant. One month after the left eye treatment, the CST in both eyes was 316 μm OD and 477 μm OS (**Figures 4A, B**), and the UCVA was 0.2 for the right eye and 0.15 for the left eye. To promote absorption of left eye edema, the patient received an intravitreal injection of conbercept (Lumitin; Chengdu Kang Hong Biotech Co, Ltd, Sichuan, China), an anti-VEGF medication (**Figures 4C, D**). One month later, the UCVA of both eyes were 0.3 OD and 0.12 OS, respectively, and the morphology of the macular structure for both eyes has returned to normal (**Figures 4E, F**). Almost one year later, the patient visited for a follow-up and complained about his left eye central scotoma, which had been appearing since the first visit. The UCVA of both eyes was 0.3, and the BCVA was 1.0 OD and 0.8 OS. The standard 24-2 and the central 10-2 visual field examination reveal no exception. The macular morphology remained normal, with CST 241 μm for the right eye and 234 μm for the left eye (**Figures 4G, H**).

## Discussion

In this case, the patient’s right eye CST was up to 756 μm which, to our knowledge, is the highest among published literature. The DEX implant was administered to the right eye to relieve the macular edema and shorten the recovery duration, preventing irreversible macular function damage from long-term macular edema. There were two reasons for administering the intravitreal DEX implant as adjuvant treatment. First, premedication of dexamethasone for taxoid chemotherapy has been proven effective in preventing the taxane-related adverse effects and has been widely practiced, though the mechanism details are unknown (5, 6). Therefore, we hypothesized that the DEX implant might also be able to relieve the side effects on the eyes. Second, the use of DEX implant for taxane-related CME has been safely implemented by *Burgos-Blasco* et al. and showed promising results without side effects (7).

After administering the DEX implant, an intense follow-up schedule was arranged for the patient to observe the macular responses to the DEX implant. The CST of the treated eye decreased steadily during two weeks of follow-up (**Figure 3A**). At the same time, the CST of the untreated eye continued to increase from 557 μm to 628 μm, though the albumin-bound paclitaxel had been discontinued for four weeks (**Figure 3B**). It should be noted that the terminal half-life of albumin-bound paclitaxel was about 27 hours long. The patient’s first visit was two weeks after his last administration of paclitaxel, which means the system remaining paclitaxel should already be depleted. The continuing elevation of CST in the untreated eye indicates the paclitaxel effects on macular function might continue to cause elevation of CMT without paclitaxel. Therefore, it is possible that for severe cases, the cessation of paclitaxel might not be sufficient to control the deterioration of macular function in a short time.

Meanwhile, compared to the untreated eye, the right eye CST was dropping slowly, which might indicate the DEX implant was taking effect. Hence the right eye was also administered the DEX implant to suppress the increasing trend. Two weeks after the administration of the DEX implant for the left eye, the CST dropped from 642 μm to 314 μm and continued to decrease after that.

Intravitreal injection of conbercept was applied for the worse eye to further accelerate the resolution of edema, but the efficacy is not evident in our case. Intravitreal anti-VEGF for taxane-related CME is still controversial in published literature (6–8). From the results, it might be unnecessary to administer conbercept but should wait for the DEX implant to take effect.

Due to the rarity of the taxane-related CME, the explicit diagnostic criteria are still absent from current published literature. According to previous case studies, we summarized the diagnostic criteria for taxane-related CME, which should include the following statement. First, the CME or visual symptom typically happens after four cycles of taxane-related chemotherapy (3). Second, the OCT examination shows a significant elevation of the bilateral central macular thickness, mainly occurring at the outer plexiform layer (OPL), and the inner plexiform layer (IPL) can also be affected, leading to scattered cystic spaces. The continuity of OPL and IPL in taxane-related CME differentiates from the disorganization of the retinal inner layers (DRIL) caused by retinal vein occlusion (RVO) or diabetic retinopathy (DR) (8). Third, the FFA reveals no evidence or minimal leakage of fluorescein, with or without late pooling of fluorescein. Fourth, other diseases with similar OCT manifestation should be ruled out, such as juvenile X-linked retinoschisis, Goldmann-Favre syndrome, niacin-related maculopathy, and retinitis pigmentosa (4). Fifth, the spontaneous resolution should be achieved in less than six months following the cessation of the taxoid chemotherapy (3).

So far, the primary and confirmed treatment for taxane-related CME remains the cessation of taxoid chemotherapy. Other adjuvant therapies, including topical NSAID, topical dorzolamide, topical steroids, and intravitreal anti-VEGF, remained controversial. The main reason is the rarity of taxane-related CME, making it impossible for large-scale research or even case series studies. All published literature on this topic is based on case studies. Another reason is that the taxane-related CME tends to spontaneously resolve after cessation of the taxoid chemotherapy, making it difficult to assess the efficacy of the adjuvant treatment. In 2020, *Burgos-Blasco* et al. published the first and only taxane-related CME treated by DEX implant (7). The patient did not discontinue albumin-bound paclitaxel after the DEX implant, and the CMT was reduced from 627 μm and 632 μm to 543 μm and 558 μm, respectively, after the DEX implant for one month. Albumin-bound paclitaxel was then discontinued, and one month later, the central thickness returned to 272μm and 265μm. However, in that case, the efficacy of DEX implant for taxane-related CME is obscured. Our study, through a single-eye trial, showed the possibility for DEX implant to be an adjuvant treatment for taxane-related CME and complements the shortcomings of the *Burgos-Blasco* report.

The detailed mechanism of taxane-related CME is still unknown. However, several authors have proposed that the direct toxicity of taxoid to müller cells is the leading cause of this condition. The taxoid compromises the ion regulation in müller cells, which results in swollen cells and cystic formation (4, 9). However, this hypothesis cannot explain the effectiveness of DEX implementation. Therefore, contradicting the former assumptions, we hypothesize that the inflammation induced by tanxoid might be one of the primary factors for the taxane-related CME. Through stabilizing the microtubule, taxane induces mitotic delay and micronuclei formation (10). The defected membrane of micronuclei will, in turn, activate the cGAS/STING pathway and stimulate macrophages as well as innate immunity, which Serpico et al. have illuminated *(* 11 *).* However, we cannot prove this hypothesis in this report. Considering the rarity of the taxane-related CME, *in vivo* experiments are needed to confirm the theory and the efficacy of the DEX implant for this condition.

There are several limitations in this case report. The primary one should be the administration of the conbercept, which should be waited longer. It also contaminated the data, making it hard to justify which therapy was responsible for macular anatomical recovery after that. The second one is that even though the follow-up schedule was arranged for the patient three days, one week, and two weeks post-operative for the right eye, the patient only followed up two weeks post-operative due to his health problem. Therefore, the OCT examination was not taken on one day and one week post-operative, losing the opportunity to capture the turning point of the curves after the right eye treatment.

However, there are also several strengths to this case report. First, the CST in the right eye was 756 μm, the highest recorded CST among published literature in taxane-related CME. With such severity, the outcome was satisfactory, in which the BCVA was recovered to 0.8 for the right eye. This indicates that the cessation of taxoid medication combined with the intravitreal DEX implant might be an effective therapeutic regimen for severe cases, preserving macular function by promoting macular edema absorption. Second, this is the first time using the cessation of taxoid medication with the intravitreal DEX implant as initial treatment for taxane-related CME and showed promising outcomes. We believe our case will provide new insight into the treatment of taxane-related CME.

## Conclusions

Combined with the cessation of taxoid therapy, the intravitreal DEX implant could be an effective adjuvant therapy to accelerate the recovery of taxane-related CME and reduce the irreversible damage to the macula.

## Data availability statement

The original contributions presented in the study are included in the article/Supplementary Material. Further inquiries can be directed to the corresponding author.

## Ethics statement

Ethical review and approval were not required for the study on human participants in accordance with the local legislation and institutional requirements. The patients/participants provided their written informed consent to participate in this study. Written informed consent was obtained from the individual for the publication of any identifiable images or data included in this article.

## Author contributions

GL, XL, and XD collected the clinical data. XL prepared the manuscript. XL and GL reviewed and revised the manuscript. All authors have read and approved the manuscript.

## Funding

This study was funded by the Chaozhou Science and Technology Program Project Foundation (NO. 2021ZC14).

## Conflict of interest

The authors declare that the research was conducted in the absence of any commercial or financial relationships that could be construed as a potential conflict of interest.

## Publisher’s note

All claims expressed in this article are solely those of the authors and do not necessarily represent those of their affiliated organizations, or those of the publisher, the editors and the reviewers. Any product that may be evaluated in this article, or claim that may be made by its manufacturer, is not guaranteed or endorsed by the publisher.
